# Lysosomal Proteases and Their Inhibitors

**DOI:** 10.3390/ijms251810070

**Published:** 2024-09-19

**Authors:** Vito Turk, Veronika Stoka

**Affiliations:** 1 Department of Biochemistry and Molecular and Structural Biology, Jožef Stefan Institute, 1000 Ljubljana, Slovenia; vito.turk@ijs.si; 2 Jožef Stefan International Postgraduate School, 1000 Ljubljana, Slovenia

The discovery of the lysosome, a major cytoplasmic organelle, represents a breakthrough in the understanding of intracellular protein degradation processes—proteolysis [1]. Lysosomes are at the crossroads of various degradation pathways such as endocytosis, autophagy, and cell death [2] and contain over fifty hydrolases. Lopez-Otin and Bond first introduced a global view of the proteolytic landscape in representative eukaryotic genomes [3]. Moreover, Brix et al. highlighted the progress and cell biological challenges of proteolysis mediated by cysteine cathepsins and legumain and addressed some myths and common questions about endo-lysosomal cysteine peptidases [4].

Cathepsins are classified into three catalytic types: serine proteases (cathepsins A and G), aspartic proteases (cathepsins D and E), and cysteine proteases (cathepsins B, C, F, H, K, L, O, S, V, X/Z, and W). Most cysteine cathepsins are well-characterized enzymes with optimal activity in a slightly acidic environment, although some of them retain their activity at a higher pH, which is important for their role outside of the endo-lysosomal system. These enzymes play a crucial role in many physiological processes such as bone resorption, hormone processing, antigen processing, and presentation, as well as in numerous pathological conditions such as inflammation-associated diseases, cancer, and neurodegenerative diseases, among others [5,6,7,8,9].

Their potentially harmful activity outside the lysosomes must be regulated by pH, zymogen activation, and protein inhibitors such as cystatins, thyropins, and others, including small-molecule synthetic inhibitors. Some lysosomal enzymes are also highly unstable at neutral pH, such as cathepsins L and B. Their inactivation is irreversible and results in the loss of their 3D structure. On the other side, some cathepsins, such as cathepsins K and S and the aspartic cathepsin D, exhibit activity at neutral pH [5].

This Special Issue consists of two original articles and three reviews, and provides further insights into this field as follows:

Kordiš and Turk [10] presented a comprehensive phylogenomic analysis tracing the early diversification and expansion of the papain family. The study found evidence of short C1A peptidases in archaea and bacteria, supporting their presence in the Last Universal Common Ancestor. Moreover, it suggested that the first eukaryotic common ancestor may have acquired C1A peptidases through horizontal gene transfer, possibly from Deltaproteobacteria. It should be noted that the transition from the first to the last eukaryotic common ancestor involved intensive reshaping of the C1A repertoire, which resulted in eight eukaryotic ancestral paralogous C1A lineages during eukaryogenesis.

Scarcella et al. 2022 [11] provided an overview of the cathepsins involved in human viral infections. They presented some representative examples of the molecular mechanisms where cathepsins support viruses’ interactions with target cells at different steps of their life cycle. The authors also discussed the potential of cathepsin inhibitors for therapeutic applications in viral infectious diseases.

Mijanović et al. 2022 [12] reviewed the role of cathepsin K as a therapeutic target resulting from a deficiency or an increased secretion of cathepsin K. In addition, the authors discussed its potential as a diagnostic biomarker and therapeutic challenges regarding the development and testing of cathepsin K inhibitors.

Some cysteine cathepsins are inhibited by such Kunitz-type inhibitors. It should be noted that an atypical and functionally diverse family of Kunitz-type cysteine/serine proteinase inhibitors secreted by the helminth parasite *Fasciola hepatica* likely regulates parasite cathepsin L proteases and/or impairs host immune cell activation [13].

Jmel et al. 2023 [14] provided a comprehensive overview of the biochemical features of tick salivary Kunitz-type protease inhibitors, emphasizing their various effects on host hemostasis and immunity at the molecular and cellular levels. Moreover, the authors discussed their potential and challenges for the development of novel therapies and vaccines. The authors summarized the discoveries and advances in Kunitz-type inhibitors since the uncovering of the family members and the major milestones in tick-derived species.

Finally, Holzner et al. 2023 [15] showed that another cysteine protease, legumain (C13D), can also exert important functions outside the lysosome, its primary localization. The work provided in vitro evidence that legumain can function as a BDNF-dependent TrkB sheddase, which suppresses TrkB-mediated pro-survival signaling in the absence of BDNF. The authors presented another mechanistic link, thus explaining the reciprocal TrkB signaling and δ-secretase activity of legumain, which is relevant for neurodegeneration.

Recent advances provide further understanding of lysosomal proteases and their endogenous or small synthetic inhibitors. Lysosomal proteases are also important diagnostic and therapeutic targets for various diseases, while their inhibitors are already in clinical trials [6,8]. Recent studies of cysteine proteases of parasites have shown that they are involved in numerous biological processes such as immune evasion, pathogenesis, and virulence and have been validated as drug targets.

Overall, this Special Issue combines original papers with review articles on current topics in the broad field of lysosomal proteases and their inhibitors. We hope that it will be valuable for both experts and newcomers in the field.
